# Predictive validity of attention-deficit/hyperactivity disorder from ages 3 to 5 Years

**DOI:** 10.1007/s00787-021-01750-5

**Published:** 2021-03-07

**Authors:** Kristin Romvig Overgaard, Beate Oerbeck, Svein Friis, Are Hugo Pripp, Heidi Aase, Pål Zeiner

**Affiliations:** 1 Division of Mental Health and Addiction, Oslo University Hospital, Pb. 4959 Nydalen, 0424 Oslo, Norway; 2 Institute of Clinical Medicine, University of Oslo, Oslo, Norway; 3 Oslo Centre of Biostatistics and Epidemiology, Oslo University Hospital, Oslo, Norway; 4 Department of Child Health and Development, Norwegian Institute of Public Health, Oslo, Norway

**Keywords:** Attention-deficit/hyperactivity disorder, Children, Longitudinal, Sensitivity

## Abstract

We investigated to what extent parent-rated attention-deficit/hyperactivity disorder (ADHD) and impairment at age 3 years predicted elevated ADHD symptoms at age 5 years, and whether teacher-rated ADHD symptoms improved these predictions. This study is part of the longitudinal, population-based Norwegian Mother, Father and Child Cohort Study. Parents of 3-year-old children (*n* = 1195) were interviewed about ADHD and impairment, and teachers rated child ADHD symptoms by the Strengths and Difficulties Questionnaire or the Early Childhood Inventory-4. At 5 years of age, the children (*n* = 957) were classified as ADHD-positive or -negative using Conners’ Parent Rating Scale. Relying solely on parent-rated ADHD or impairment at age 3 years did moderately well in identifying children with persistent elevation of ADHD symptoms, but gave many false positives (positive predictive values (PPVs): .40–.57). A small group of children (*n* = 20, 13 boys) scored above cut-off on both parent-rated ADHD and impairment, and teacher-rated ADHD symptoms, although adding teacher-rated ADHD symptoms slightly weakened the predictive power for girls. For this small group, PPVs were .76 for boys and .64 for girls. Limiting follow-up to these few children will miss many children at risk for ADHD. Therefore, we recommend close monitoring also of children with parent-reported ADHD symptoms and/or impairment to avoid delay in providing interventions. Clinicians should also be aware that teachers may miss ADHD symptoms in preschool girls.

Attention-deficit/hyperactivity disorder (ADHD) is characterized by age-inappropriate and impairing hyperactivity-impulsivity and/or inattention that is pervasive across settings [1]. ADHD often begins during preschool years [1] and occurs 1.6–1.8 times more frequently in preschool boys than girls [2, 3] with a preschool prevalence rate of 1.9–3.3% when estimated by a parent diagnostic interview [3, 4]. Studies report that greater symptom severity in early childhood predicts poor outcomes [5–8]; therefore, early detection is recommended [9]. Furthermore, during the last 20 years, prospective clinical studies have found strong predictive validity, where ≥ 70% of those who met criteria for ADHD from the age of 4 years old and over continued to fulfil criteria at 6- and 8-year follow-ups [5, 7, 10, 11]. Subsequently, the American Academy of Pediatrics extended their guidelines for the ADHD diagnosis to apply from the age of 4 years [9]. However, the fifth edition of the *Diagnostic and Statistical Manual of Mental Disorders* (DSM-5) states that ADHD symptoms are difficult to distinguish from normative behaviours before this age [1].

Still, the few longitudinal studies that begin before the age of 4 years indicate that ADHD symptoms at this early age may be important to identify [12]. One community study found that ADHD diagnosed at the age of 3 years gave a significantly increased odds ratio (OR = 17.96) of ADHD at 6 years old [13], but was limited by the low number of children diagnosed with ADHD at age 3 years (*n* = 11). Another community study with 170 hyperactive 3-year-old children found significant continuity between early hyperactivity and ADHD after 14–25 years, but only in boys [14]. Another study including 3-year-old children with behavioural problems (*n* = 168), found that 58% of the children with behavioural problems continued to meet diagnostic criteria 3 years later [15]. None of these studies included impairment or used the recommended multi-informant, across-settings approach to assess ADHD at age 3 years [1, 9, 16]. However, one clinical study followed children (baseline, mean age = 5.2 years) with ADHD who were impaired in one (*n* = 96) or two (*n* = 29) settings (according to parent or teacher reports) annually for 3 years [17]. Both groups had increased risk of continuing to fulfil diagnostic criteria, and those who were impaired in one setting only had significantly lower risk than children with impairment across settings. Another study of hyperactive/inattentive pre-schoolers (*n* = 104, baseline mean age = 4.4 years) found that the predictive utility of parent-reported ADHD symptoms (≥ 90th percentile on the ADHD rating scale) was significantly improved by the addition of teacher reports [18]. Again, these two studies included older preschool children, were limited by relatively few participants, and did not investigate the potential biases when parents and teachers rated boys’ and girls’ behaviour [19]. As mentioned above, the one study that investigated gender differences from the age of 3 years, found preschool hyperactivity to be a risk factor for long-term ADHD only in boys [14]. Given the assumed referral biases of clinical preschool studies, population-based longitudinal studies on very young children, have long been in demand [20].

In the present study, we followed a cohort of 3-year-old children until they were 5 years old (*n* = 957). Based on the diagnostic criteria [1] and data from the few studies on pre-schoolers, we hypothesized that the predictive utility of ADHD classified by the parent interview when the child was 3 years old would be significantly improved by the addition of impairment and teacher-rated ADHD symptoms. As studies of possible gender differences related to the predictive validity of ADHD symptoms, impairment and pervasiveness are limited, we had no a priori hypotheses about gender differences.

## Methods

### Participants

The Norwegian Mother, Father and Child Cohort Study (MoBa) is an ongoing prospective population-based cohort study of Norwegian-speaking pregnant women conducted by the Norwegian Institute of Public Health (41% participation rate) [21]. About 89% of the participating mothers were ethnic Norwegian, recruited from all over Norway from 1999 to 2008, and were predominantly white Caucasians [22]. The current paper is from a clinical substudy on ADHD described in previous publications [23, 24]. In short, this study oversampled children at risk for ADHD using the MoBa questionnaire for 3-year-old children, which includes 11 items about ADHD; six items from the Child Behavior Checklist/1.5–5 (Can’t concentrate, Can’t sit still, Can’t stand waiting, Demands must be met immediately, Gets into everything, Quickly shifts activities) [25], and five items from the revised fourth edition of the DSM (DSM-IV-TR) (Easily distracted, Difficulty waiting his/her turn, Difficulty sustaining attention, Talks excessively, Does not seem to listen) [26]. Children with scores ≥ 90th percentile on these 11 items (*n* = 2798) were invited to participate, along with randomly selected children from MoBa (*n* = 654). Of these, 35% agreed to participate, and 1195 children took part in a 1-day clinical assessment that included a diagnostic interview with parents (almost all mothers) from 2007 to 2011. Ninety-five percent of Norwegian 3-year-olds attend preschool. When the children were 3 years old, their parents received the screening questionnaires by mail and gave theirs to the teachers, who mailed their responses to the study administrator. By the 5-year follow-up, 6 mothers withdrew from the MoBa-study, leaving 1,189 participants. Eighty percent (*n* = 957; 519 boys) of the mothers who participated when their child was 3 years old also completed questionnaires when their child was 5 years old.

## Measures

### Background variables

Length of parental (mothers’ + fathers’) education was obtained at the first MoBa assessment (about Week 17 of pregnancy) and reported in mean number of years.

To check how many children were referred for treatment, we included results on one item asked to mothers at child age 3 and 5 years: “Has your child been referred to Child and Adolescent Mental Health Services (CAMHS)? To check for comorbidities at age 5 years, we included two questions: (1) “Has your child ever had behavioural problems (difficult or oppositional behaviour)?” (2) “Has your child ever had emotional problems (sadness or anxieties)?”

#### At 3 years of age

Birth date and child gender were obtained from the Norwegian Medical Birth Registry.

The preschool age psychiatric assessment (PAPA) [27] was developed for children from 2 to 5 years old. The PAPA is interviewer-based, i.e., the interviewer asks questions until s/he can decide whether the symptoms described meet the definitions provided in a glossary. In a study of test–retest reliability of the PAPA interview, the test–retest intra-class correlations for ADHD classification was 0.80 [4]. In the present study, only ADHD symptoms persisting for ≥ 3 months were counted as present. Blinded to the questionnaire ratings, trained graduate psychology students conducted the interviews under the supervision of specialists in child psychology/psychiatry. An interrater reliability check was carried out by a second blind rater who rescored audiotapes of 79 randomly selected interviews. The average intra-class correlation for ADHD symptoms was 0.98.

#### Parents’ classifications

In line with our earlier studies [23, 24], we used information from the PAPA interview, and defined ADHD by the DSM-IV-TR criteria [26], classifying the ADHD hyperactive/impulsive or inattentive presentations by the presence of at least six out of nine symptoms of either hyperactivity/impulsivity or inattention, and ADHD combined presentation by six out of nine symptom within both symptom presentations. In all, 172 children fulfilled the criteria: 76% hyperactive/impulsive (*n* = 131, 50 girls), 5% inattentive (*n* = 8, 6 girls), and 19% combined presentation (*n* = 33, 14 girls).

The ADHD section of the PAPA concluded with an impairment section [28]. Whenever a parent reported at least one ADHD symptom, impairment was scored for six functional domains: (1) family relationships; (2) friends; (3) learning; (4) play/leisure activities; (5) child’s quality of life; and (6) family burden. For each domain, difficulties were scored on a four-point Likert scale (no, a little, some, a great deal; range 0–3). The functional domains gave a total impairment scale score (range 0–18), with Cronbach’s ɑ = 0.83. A sum score of three was equivalent to the 75th percentile for the participants who responded to the questionnaire at 5 years, in line with an earlier study of all participants at age 3 years old, and was taken to indicate a moderate level of impairment [28]. Thus, an impairment score of ≥ 3 was chosen as the threshold for impairment to be present. Among the children who fulfilled ADHD symptom criteria according to the PAPA interview, impairment was present in 62% of boys and 50% of girls.

Oppositional Defiant Disorder (ODD) was defined by the presence of ≥ 4 DSM-IV ODD symptoms according to PAPA.

#### Teachers’ classifications

The first 44% of the sample received the Norwegian version of the Strengths and Difficulties Questionnaire (SDQ) rated on a three-point Likert scale (not true, somewhat true, and certainly true; range 0–2) [29]. The psychometric properties of the teacher SDQ have been found satisfactory [30]. We used only the hyperactivity/inattention (HI) subscale (Cronbach’s ɑ = 0.86), which we in a previous cross-sectional study at age 3 years found to discriminate PAPA classified ADHD from non-ADHD significantly better than chance (*p* < 0.001) [23]. We used a cut-off of seven to categorize ADHD, in line with epidemiological studies [31–34].

The rest of the sample received the Norwegian version of the Early Child Inventory-4 (ECI-4) that contains 108 items corresponding to the symptom lists of the child psychiatric disorders in the DSM-IV [35]. The inventory was translated into Norwegian and back-translated into English by professional translators, and the translation was approved by the ECI-4 developers. We used only the teacher hyperactivity/impulsivity (H-I) and inattention (IA) subscales, each with nine items [35] rated on a four-point Likert scale (never, sometimes, often, very often; range 0–3). In a previous cross-sectional study, at age 3 years, we found these teacher-rated subscales to discriminate PAPA classified ADHD from non-ADHD significantly better than chance (*p* < 0.001) [24]. We used the dichotomized teacher symptom count where symptoms were scored as being not present (never/sometimes = 0) or present (often/very ofte* n* = 1). In line with the ECI-4 manual, the cut-off score was set to the minimum number of symptoms necessary for the DSM-IV ADHD diagnosis (≥ 6 on either the H-I or the IA subscales) [36]. Cronbach’s α was 0.91/0.90 for the H-I/IA subscales. Pearson’s correlation between the two subscales was high (0.78; *p* < 0.001).

Thus, for the entire sample, teacher ADHD was classified as present by an SDQ HI score ≥ 7 (first subsample) or by an ECI-4 H-I or IA symptom count score ≥ 6 (last subsample).

#### At 5 years of age

The mothers received the revised short form of the Conners’ Parent Rating Scale (CPRS-R:S) including the 12 ADHD items comprising the ADHD index [37]. The CPRS items were rated on a four-point Likert scale (not true, somewhat true, often true, or very true; range 0–3), with Cronbach’s α = 0.88. The ADHD index has been found to have good validity for ADHD [38]. From all the responders to the five-year MoBa questionnaire (*n* = 32,633, 49% girls), we created cut-offs for ADHD to be present at the 95th percentile of the CPRS-R:S ADHD index scores: > 14.2 for boys and > 11 for girls. In our sample, 215 children (121 boys, 94 girls) reached the ADHD classification by these cut-offs, giving a 22.5% (215/957) occurrence rate.

As mentioned, only eighty percent (*n* = 957) completed the CPRS-R:S ADHD index when their child was 5 years old, we therefore checked for differences in rates of ADHD among responders and non-responders. Of the missing participants (*n* = 232), 22% of children fulfilled symptom criteria for ADHD at 3 years old, which is nearly the same as for those who continued to participate (22.5%). However, the non-responders had significantly higher parent-reported ADHD symptom scores at 3 age years old (mean = 4.76, SD = 3.96) than the responders (mean = 3.84, SD = 3.83; *t* = 3.25, *p* = 0.001), while there was no significant difference in teacher-reported ADHD symptoms. The parents of the responders were significantly better educated (mean = 14.97 years, SD = 2.20) than the parents of non-responders (mean = 14.47, SD = 2.29, *t* = 2.91, *p* = 0.004). There were more boys (54%) than girls among the responders, and vice versa among the non-responders (45% boys), a significant difference (χ 2 = 6.62, *p* = 0.01). At age 3 years, only 16 children (1.3%) had been referred to CAHMS. All inter-correlation coefficients between the parent and teacher variables when the child was 3 years old and CPRS-R:S when the child was 5 years old were low to moderate (range 0.27–0.61) (Table S1).

### Ethics

Informed consent was obtained from participants upon recruitment to the MoBa and the ADHD substudy. Both studies had licenses from the Norwegian Data Inspectorate. The present study was approved by the Regional Committee for Medical Research Ethics.

### Analytic plan

Internal consistencies were analysed using Cronbach’s α, differences between means of continuous variables with *t* tests and correlations with Pearson’s r. For the defined categories (parent-rated ADHD, impairment, and teacher-rated ADHD), we estimated sensitivity (the probability that a measure correctly classified a child with ADHD at age 5 years) and specificity (the probability that a measure correctly identified a child without ADHD at age 5 years). We calculated the positive predictive values (PPVs, the probability of a child being correctly classified with ADHD at age 5 years given a positive test), negative predictive values (NPVs, the probability of a child being correctly classified without ADHD at age 5 years given a negative test), positive likelihood ratios (+ LRs; the probability of a child being classified with ADHD at age 5 years testing positive divided by the probability of a child being classified without ADHD at age 5 years testing positive), and negative LRs (–LRs; the probability of a child being classified with ADHD at age 5 years testing negative divided by the probability of a child being classified without ADHD at age 5 years testing negative). LRs between 0 and 1 argue against the presence of the disorder [39]. Because LRs are derived from sensitivity and specificity and are independent of the proportion of the disorder in the sample, they are more likely than PPV/NPV to generalize results outside the sample [40].

## Results

Children classified with ADHD at 5 years had significantly higher mean parent and teacher symptom scores at 3 years than those who were not classified with ADHD (Table 1).

**Table 1:** *t*-test Comparisons of the 3-Year-Old Measures for Boys and Girls Categorized With and Without ADHD at 5 Years Old

	n	ADHD 5 Years Mean (SD)	n	No ADHD 5 Years Mean (SD)	*t*-test	*p*-value
Boys, 3 years (*n* = ADHD/no ADHD)	121	54/67	398	48/350		
p-PAPA ADHD symptoms	121	7.17 (3.97)	398	3.20 (3.33)	9.97	< .001
p-PAPA impairment scores	121	3.21 (3.69)	398	1.18 (2.10)	5.77	< .001
t-SDQ HI	44	5.43 (2.73)	182	3.64 (2.72)	3.92	< .001
t-ECI H-I	66	8.94 (6.59)	191	5.64 (4.88)	3.73	< .001
t-ECI IA	65	8.57 (6.09)	185	5.51 (4.22)	3.75	< .001
Girls, 3 years (*n* = ADHD/no ADHD)	94	40/54	344	30/314		
p-PAPA ADHD symptoms	94	6.97 (2.56)	344	2.54 (2.97)	9.39	< .001
p-PAPA impairment scores	94	2.81 (3.04)	344	.80 (1.41)	6.22	< .001
t-SDQ HI	37	4.30 (2.99)	149	2.49 (2.47)	3.91	< .001
t-ECI H-I	53	6.11 (4.38)	174	4.12 (4.11)	3.73	< .001
t-ECI IA	53	6.30 (5.58)	173	3.77 (4.22)	3.75	< .001

*p* parent, *t* teacher, *SDQ* Strengths and Difficulties Questionnaire, *HI* Hyperactivity Inattention subscale, *ECI-4* Early Childhood Inventory-4, *H-I* Hyperactivity-Impulsivity subscale, *IA* Inattention subscale, *CPRS-R* S Conners’ Parent Rating Scale

ADHD at 5 years was classified as ≥ 95th percentile on the CPRS-R:S ADHD index sum scores in the MoBa population (> 14.2 for boys and > 11 for girls)

The parent-classified ADHD and impairment at 3 years gave similar sensitivity (43–45%) and specificity values (81–91%) for detecting elevated ADHD symptoms at 5 years old in boys and girls. In boys, the teacher-classified ADHD gave sensitivity and specificity values of 27% and 88%, respectively, while the sensitivity of the teacher classification was particularly low (14%) in girls. For boys, the highest + LR of being classified with ADHD at 5 years was found for the children with parent-rated ADHD, impairment, and teacher-rated ADHD (+ LR = 10.79), but the sensitivity was low (12%) for this combination. For girls, the combination of the parent-rated ADHD and impairment gave a + LR of 7.98 and adding teacher-rated ADHD symptoms slightly weakened the predictive power (see Table 2). Only a small group of 3-year-old children (13 boys, 7 girls) fulfilled ADHD criteria according to both parent and teacher reports, and were impaired. These children had high probabilities of being categorized with elevated ADHD symptoms at 5 years (PPVs = 0.76 in boys, 0.64 in girls).

**Table 2:** Prediction Values for ADHD Classified at 5 Years Old

Predictors, 3 years	N/n*	Se (%)	Sp (%)	PPV	NPV	+ LR	–LR
Boys							
Parent-rated ADHD	519/54	45	88	.53	.84	3.70	.63
Parent-rated impairment	519/53	44	81	.42	.83	2.36	.69
Teacher-rated ADHD	488/31	27	88	.40	.80	2.24	.83
Parent-rated ADHD and impairment	519/35	29	93	.57	.81	4.43	.76
Parent-rated ADHD, impairment, and teacher-rated ADHD	488/13	12	99	.76	.79	10.79	.89
Girls							
Parent-rated ADHD	438/40	43	91	.57	.85	4.88	.63
Parent-rated impairment	438/42	45	88	.49	.85	3.57	.63
Teacher-rated ADHD	417/13	14	94	.42	.80	2.55	.91
Parent-rated ADHD and impairment	438/24	26	97	.69	.83	7.98	.77
Parent-rated ADHD, impairment, and teacher-rated ADHD	417/7	08	99	.64	.79	6.18	.94

Parent-rated ADHD (DSM-IV criteria) and impairment (scores ≥ 3) were classified by PAPA. Teacher-rated ADHD was measured using the Strengths and Difficulties Questionnaire hyperactivity/inattention subscale scores ≥ 7 or the Early Childhood Inventory-4 hyperactivity/impulsivity/inattention subscale scores ≥ 6

ADHD at 5 years was classified as ≥ 95th percentile on the Conners’ Parent Rating Scale (CPRS-R:S) ADHD index sum scores in the MoBa population (> 14.2 for boys and > 11 for girls). The proportion of children who fulfilled ADHD criteria in the sample according to the CPRS-R:S was 22.5%

*Se* Sensitivity, *Sp* specificity, *PPV/NPV* positive/negative predictive values, * + LRs/–LRs* positive/negative likelihood ratios

^*^n = Total number of boys and girls with the measure, *n* = number of true positives according to the CPRS-R:S

Overall, adjusting the thresholds for the different teacher measures at 3 years did not improve predictions.

**Fig. 1:**
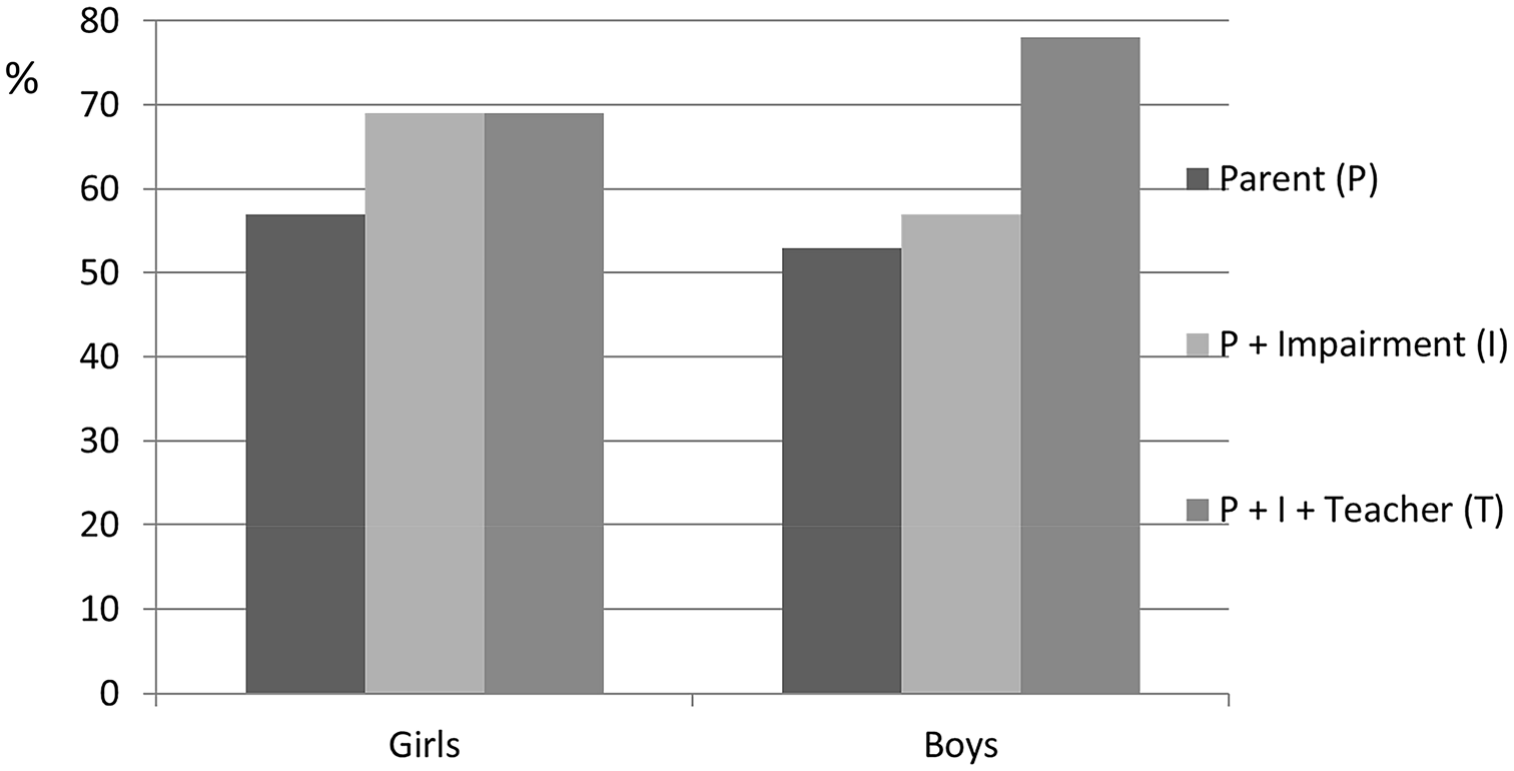
Percentages of the positive predictive values for boys and girls classified with ADHD at 5 years for the 3-year categories: parent-classified ADHD (P), parent-classified ADHD and impairment (I), and parent-classified ADHD, impairment, and teacher-classified ADHD (T)

**Fig. 2:**
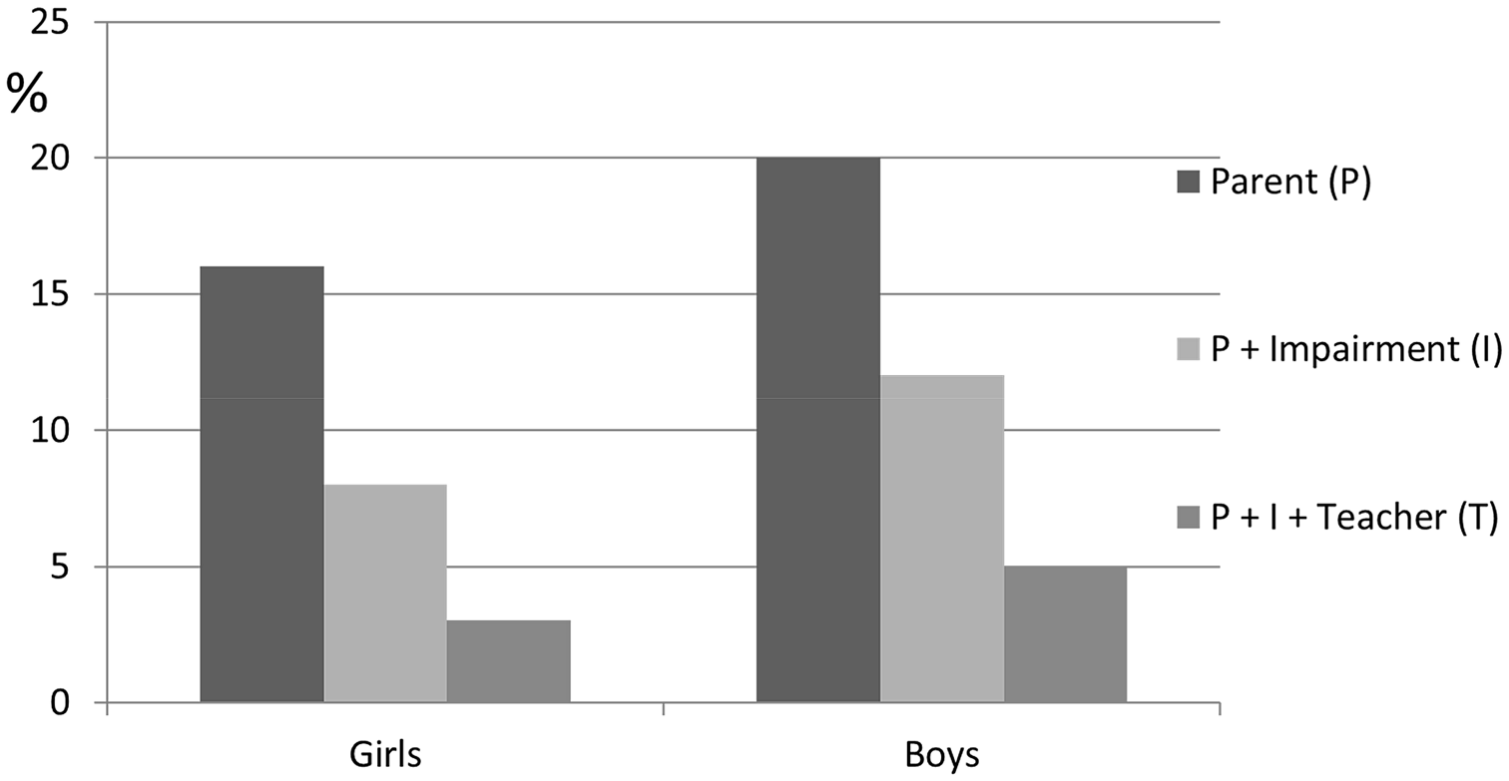
Percentages of the boys and girls who met the criteria for ADHD at 3 years according to three different categories: parent-classified ADHD (P), parent-classified ADHD and impairment (I), and parent-classified ADHD, impairment, and teacher-classified ADHD (T)

Figure 1 illustrates the improved PPVs in boys when impairment and teacher assessments were added to parent scores, with an increase from 53 to 76%. This effect was not found in girls. However, the highest PPVs were only valid for a small group of children. By demanding a combination of criteria, the percentage of children classified with ADHD at 3 years dropped dramatically (Fig. 2).

ODD at age three gave a sensitivity of 53% for detecting ADHD at age 5 years in both genders, with corresponding specificity values of 71% and 66%, and + LRs of 1.83 and 1.55, in boys and girls, respectively. Adding ODD to the parent ADHD, impairment and/or teacher ADHD did not improve detection of ADHD classified at age 5 years.

## Discussion

Consistent with our hypothesis, the predictive power improved when impairment and teacher-rated ADHD were added to parent-rated ADHD, but only in boys. Relying on only one source of information to find children categorized with ADHD at 5 years old gave much uncertainty due to many false positives (PPVs = 0.40–0.57). In the small group of 3-year-old children (13 boys, 7 girls) who fulfilled ADHD criteria according to both parent and teacher reports, and were impaired, the probability of finding children categorized with ADHD at 5 years was high (PPVs = 0.76 in boys, 0.64 in girls). For girls, the highest PPV (0.69) was reached by combining information about parent-rated ADHD and impairment.

Our finding of increased risk for persistent elevation in ADHD symptoms even in children with only parent-rated symptoms and/or impairment is in accordance with a community study that found that children diagnosed with ADHD by parent interview at 3 years old were highly likely to meet ADHD criteria at 6 (OR = 17.96), 9 (OR = 5.53), and 12 years old (OR = 6.80) [13, 41]. That study only identified 11 pre-schoolers with ADHD at the age of 3 years old (about 2%), and reported increased occurrence rates at school age (13.1% and 16.4% at ages 9 and 12 years, respectively) [41]. This increase may have been due to ADHD symptoms becoming more apparent in school. It might also be that the use of telephone interviews when the children were 3 years old did not detect all children who had ADHD already at that age [41]. The increased occurrence rates in that study may have inflated their ORs, in contrast with our study where about 20% were categorized with ADHD at both time points.

The moderate diagnostic stability of ADHD when relying on one source of information is not unique for 3-year-old children. As pointed out by Law et al., community samples using checklists to ascertain ADHD diagnoses have reported about 50% stability in < 7-year-old children after a 2-year period [7]. Studies of clinical/high-risk samples that include some form of standardized diagnostic assessments have demonstrated higher stability (> 70%), suggesting that more narrow study selections and broader clinical assessments improve prediction [7, 10, 15]. This may explain why the stability for the few children with parent-rated ADHD, impairment, and teacher-rated ADHD in the present study was similar to that of children in these clinical studies. However, limiting follow-up to these few children will miss many children at risk for ADHD. Therefore, we recommend close monitoring of children with parent-reported ADHD symptoms and/or impairment, in line with a recent review emphasizing that pre-schoolers with subthreshold ADHD are more likely to develop the disorder over the next few years than to outgrow it [12].

Impairment in the present study had similar sensitivity and specificity values to the ADHD symptoms measures, in accordance with a study of school children that found impairment of ADHD at baseline significantly predicted persistence of ADHD 11 years later [42]. Our finding was also consistent with that of a preschool study, which found that impairment gave an increased risk of fulfilling diagnostic criteria over the next 3 years [17].

As in previous studies [43–46], we found low to moderate correlations between parent- and teacher-rated ADHD symptoms in 3-year-old children. An earlier study underlined the importance of a multi-informant approach, showing that the predictive power of parent-reported ADHD was significantly improved by adding teacher reports [18]. This was consistent with our results only for boys because teacher-rated ADHD symptoms had a particularly low sensitivity for girls (14%). This gender difference contrasts to our earlier finding that preschool teachers were inaccurate in identifying 3 years old boys at risk, compared with a parent diagnostic interview [23], and also with a follow-up study of school-children reporting that the combination of parent and teacher checklist information improved predictive power most clearly in girls [47].

The strengths of our study included the population-based cohort design and the use of a parent diagnostic interview when the children were 3 years old, but it also had limitations. There were selection biases due to attrition [21, 48], however, one MoBa-study reporting on ADHD found differences to be small and assumed limited effects on generalizability [49]. We specifically checked for biases in the present study, and found the parents of the responders significantly better educated than the parents of non-responders, as well as more boys (54%) than girls among responders, and vice versa among non-responders (45% boys). Although these differences were statistically significant, they were small, indicating that attrition at 5 years has not biased our results substantially. Also, we found significantly higher parent-reported ADHD symptom scores at 3 years old among non-responders than the responders, indicating that if the non-responders had been retained, the prediction of ADHD at age 5 years might have been stronger, at least for parent-reported symptoms and impairment. About 1% and 5% of the sample had been referred to CAHMS before ages 3 and 5 years, respectively. Among the children classified with ADHD according to Conners at 5 years, about 14% (*n* = 31) had been referred to CAHMS, significantly more than among the children not classified with ADHD at age 5, of whom 2% (*n* = 17) had been referred. Although we cannot entirely rule out that treatments received may have influenced whether the children continued to fulfil criteria at age 5 years, few had been referred, particularly for those who were not classified with ADHD at 5 years. Also, according to the Norwegian prescription database, very few children in Norway are medicated for ADHD before age 5 years, with a variation in medication rate from 0.02/1000 to 0.1/1000 during a fifteen-year period (2004—2019) (http://www.norpd.no/). These rates are in line with a study from the five Nordic countries reporting annual prevalence of ADHD drug use under age 6 years in 1/1000 boys and 0.3/1000 girls [50]. About 9% and 5% of the sample were reported to have had behavioural or emotional problems, respectively. As expected, there were significantly more children with a life-time history of these problems among children classified with ADHD compared to those not classified with ADHD at age 5 years (behavioural: 24% versus 4%; (*p* < 0.001); emotional: 12% versus 3% (*p* < 0.001), in line with studies reporting high comorbidity rates to ADHD [51, 52]. Our diagnostic outcome was based solely on the CPRS ADHD index scores, and is not equivalent to clinical ADHD diagnoses, but this index was found to have good validity for ADHD [38]. We created cut-offs based on the ADHD 95th percentile scores in the large MoBa population, indicating that we may have a group of high ADHD scorers compared with their peers. Also, using a cut-off based on scores in the general population from which the children were drawn, reduces the problem of cross-cultural differences if using US cut-off scores, as discussed in a previous paper [24]. Unfortunately, we only had parent-reported ADHD symptoms at age 5 years and cannot rule out that teacher-reported ADHD at this age might have altered our results somewhat.

There was a larger proportion of girls than boys with ADHD inattentive presentation at age three (6 girls of 8 children), but most of the children had hyperactive/impulsive or combined presentations (91% girls and 98% boys). Therefore, this gender difference can hardly explain why teachers were poor at identifying girls later categorized with ADHD. Because most of our sample had the hyperactive/impulsive presentation at age 3 years (76%), and we only had CPRS ADHD index at age 5, we were unable to investigate ADHD subtype stability/instability. Likewise, we did not fully address the role of comorbidity for this stability/instability, but we found that ODD, the most common comorbid disorder to ADHD, did not improve prediction of ADHD classified at age five years. Our at-risk sample had a higher proportion of children classified with ADHD at 5 years (22.5%) than did the general population, increasing the probability of finding true cases given a positive screen result. However, this should not affect LR-estimates, which may generalize to other samples.

## Implications and future directions

In the present study, children where only the parent-reported child ADHD symptoms or impairment at age 3 years constitute a high-risk group, as about 50% continued to be categorized with ADHD at age 5 (according to parent report). We also identified a few children with combined parent- and teacher-reported ADHD and impairment with high probability of persistent elevation of ADHD symptoms to age 5 years. However, limiting follow-up to these few children will miss many children at risk for ADHD. Therefore, we recommend close monitoring also of children with parent-reported ADHD symptoms and/or impairment to avoid delay in providing interventions, in line with ADHD guidelines [9]. Clinicians should also be aware that preschool teachers may have more difficulty detecting ADHD in girls than in boys. Future studies should investigate whether the predictive validity of preschool subthreshold ADHD will be as likely as above-threshold ADHD to develop into ADHD at school age. The gender differences in the predictive validity of teacher reports in the present study need to be replicated.

## References

[1] American Psychiatric Association (APA) (2013). Diagnostic and statistical manual of mental disorders: DSM-5.

[2] Willcutt EG (2012). The prevalence of DSM-IV attention-deficit/hyperactivity disorder: a meta-analytic review. Neurotherapeutics.

[3] Wichstrom L, Berg-Nielsen TS, Angold A, Egger HL, Solheim E, Sveen TH (2012). Prevalence of psychiatric disorders in preschoolers. J Child Psychol Psychiatry.

[4] Egger HL, Erkanli A, Keeler G, Potts E, Walter BK, Angold A (2006). Test-retest reliability of the preschool age psychiatric assessment (PAPA). J Am Acad Child Adolesc Psychiatry.

[5] Lahey BB, Pelham WE, Loney J, Lee SS, Willcutt E (2005). Instability of the DSM-IV subtypes of ADHD from preschool through elementary school. Arch Gen Psychiatry.

[6] Lahey BB, Lee SS, Sibley MH, Applegate B, Molina BSG, Pelham WE (2016). Predictors of adolescent outcomes among 4–6-year-old children with attention-deficit/hyperactivity disorder. J Abnorm Psychol.

[7] Law EC, Sideridis GD, Prock LA, Sheridan MA (2014). Attention-deficit/hyperactivity disorder in young children: predictors of diagnostic stability. Pediatrics.

[8] Rajendran K, O'Neill S, Marks DJ, Halperin JM (2015). Latent profile analysis of neuropsychological measures to determine preschoolers' risk for ADHD. J Child Psychol Psychiatry.

[9] Subcommittee on Attention-Deficit, Hyperactivity D, Steering Committee on Quality I, Management, (2011). ADHD: clinical practice guideline for the diagnosis, evaluation, and treatment of attention-deficit/hyperactivity disorder in children and adolescents. Pediatrics.

[10] Riddle MA, Yershova K, Lazzaretto D (2013). The Preschool attention-deficit/hyperactivity disorder treatment study (PATS) 6-year follow-up. J Am Acad Child Adolesc Psychiatry.

[11] Lahey BB, Hartung CM, Loney J, Pelham WE, Chronis AM, Lee SS (2007). Are there sex differences in the predictive validity of DSM-IV ADHD among younger children?. J Clin Child Adolesc Psychol.

[12] Halperin JM, Marks DJ (2019). Practitioner review: assessment and treatment of preschool children with attention-deficit/hyperactivity disorder. J Child Psychol Psychiatry.

[13] Bufferd SJ, Dougherty LR, Carlson GA, Rose S, Klein DN (2012). Psychiatric disorders in preschoolers: continuity from ages 3 to 6. Am J Psychiatry.

[14] Smith E, Meyer BJ, Koerting J (2017). Preschool hyperactivity specifically elevates long-term mental health risks more strongly in males than females: a prospective longitudinal study through to young adulthood. Eur Child Adolesc Psychiatry.

[15] Harvey EA, Youngwirth SD, Thakar DA, Errazuriz PA (2009). Predicting attention-deficit/hyperactivity disorder and oppositional defiant disorder from preschool diagnostic assessments. J Consult Clin Psychol.

[16] World Health Organization (WHO). *International Classification of Diseases and related health problems (ICD-10).* 10 ed. Geneva: World Health Organization; 1990.

[17] Lahey BB, Pelham WE, Loney J (2004). Three-year predictive validity of DSM-IV attention deficit hyperactivity disorder in children diagnosed at 4–6 years of age. Am J Psychiatry.

[18] O'Neill S, Schneiderman RL, Rajendran K, Marks DJ, Halperin JM (2014). Reliable ratings or reading tea leaves: can parent, teacher, and clinician behavioral ratings of preschoolers predict ADHD at age six?. J Abnorm Child Psychol.

[19] Gershon J (2002). A meta-analytic review of gender differences in ADHD. J Atten Disord.

[20] Wilens TE, Biederman J, Brown S, Monuteaux M, Prince J, Spencer TJ (2002). Patterns of psychopathology and dysfunction in clinically referred preschoolers. J Dev Behav Pediatr.

[21] Magnus P, Birke C, Vejrup K (2016). Cohort profile update: the Norwegian mother and child cohort study (MoBa). Int J Epidemiol.

[22] Mellingen S, Torsheim T, Thuen F (2013). Changes in alcohol use and relationship satisfaction in Norwegian couples during pregnancy. Subst Abuse Treat Prev Policy.

[23] Overgaard KR, Oerbeck B, Friis S (2018). Attention-deficit/hyperactivity disorder in preschoolers: the accuracy of a short screener. J Am Acad Child Adolesc Psychiatry.

[24] Overgaard KR, Oerbeck B, Friis S (2019). Screening with an ADHD-specific rating scale in preschoolers: a cross-cultural comparison of the Early Childhood Inventory-4. Psychol Assess.

[25] Achenbach TM, Rescorla L (2010). Multicultural Supplement to the Manual for the ASEBA Preschool Forms & Profiles: Child Behavior Checklist for ages 1 1/2-5, Language Development Survey, Caregiver-Teacher Report Form : An Integrated System of Multi-Informant Assessment.

[26] American Psychiatric Association (APA) (2000). Diagnostic and statistical manual of mental disorders: DSM-IV-TR.

[27] Egger HL, Angold AC, DelCarmen-Wiggins R, Carter A (2004). The preschool age psychiatric assessment (PAPA): a structured parent interview for diagnosing psychiatric disorders in preschool children. Handbook of Infant, Toddler, and Preschool Mental Health Assessment.

[28] Bendiksen B, Aase H, Svensson E (2014). Impairment in young preschool children with symptoms of attention-deficit/hyperactivity disorder and co-occurring oppositional defiant disorder and conduct disorder. Scand J Child Adolesc Psychiatry Psychol.

[29] Goodman R (1997). The strengths and difficulties questionnaire: a research note. J Child Psychol Psychiatry.

[30] Stone LL, Otten R, Engels RC, Vermulst AA, Janssens JM (2010). Psychometric properties of the parent and teacher versions of the Strengths and Difficulties Questionnaire for 4- to 12-year-olds: a review. Clin Child Fam Psychol Rev.

[31] Cuffe SP, Moore CG, McKeown R (2009). ADHD and health services utilization in the National Health Interview Survey. J Atten Disord.

[32] Cuffe SP, Moore CG, McKeown RE (2005). Prevalence and correlates of ADHD symptoms in the national health interview survey. J Atten Disord.

[33] Dopfner M, Breuer D, Wille N, Erhart M, Ravens-Sieberer U, group Bs, (2008). How often do children meet ICD-10/DSM-IV criteria of attention deficit-/hyperactivity disorder and hyperkinetic disorder? Parent-based prevalence rates in a national sample–results of the BELLA study. Eur Child Adolesc Psychiatry.

[34] Ullebo AK, Posserud MB, Heiervang E, Gillberg C, Obel C (2011). Screening for the attention deficit hyperactivity disorder phenotype using the strength and difficulties questionnaire. Eur Child Adolesc Psychiatry.

[35] Gadow KD, Sprafkin J (2000). Early childhood inventory-4: screening manual.

[36] Gadow KD, Sprafkin J (1997). Early childhood inventory-4: norms manual.

[37] Conners CK, Sitarenios G, Parker JD, Epstein JN (1998). The revised Conners' parent rating scale (CPRS-R): factor structure, reliability, and criterion validity. J Abnorm Child Psychol.

[38] Kumar G, Steer RA (2003). Factorial validity of the Conners' Parent Rating Scale-revised: short form with psychiatric outpatients. J Pers Assess.

[39] McGee S (2002). Simplifying likelihood ratios. J Gen Intern Med.

[40] Youngstrom EA (2014). A primer on receiver operating characteristic analysis and diagnostic efficiency statistics for pediatric psychology: we are ready to ROC. J Pediatr Psychol.

[41] Finsaas MC, Bufferd SJ, Dougherty LR, Carlson GA, Klein DN (2018). Preschool psychiatric disorders: homotypic and heterotypic continuity through middle childhood and early adolescence. Psychol Med.

[42] Biederman J, Petty CR, Clarke A, Lomedico A, Faraone SV (2011). Predictors of persistent ADHD: an 11-year follow-up study. J Psychiatr Res.

[43] Korsch F, Petermann F (2014). Agreement between parents and teachers on preschool children's behavior in a clinical sample with externalizing behavioral problems. Child Psychiatry Hum Dev.

[44] Graves SL, Blake J, Kim ES (2012). Differences in parent and teacher ratings of preschool problem behavior in a national sample: The significance of gender and SES. Journal of Early Intervention.

[45] Brown JD, Wissow LS, Gadomski A, Zachary C, Bartlett E, Horn I (2006). Parent and teacher mental health ratings of children using primary-care services: interrater agreement and implications for mental health screening. Ambul Pediatr.

[46] Dirks MA, Boyle MH, Georgiades K (2011). Psychological symptoms in youth and later socioeconomic functioning: do associations vary by informant?. J Clin Child Adolesc Psychol.

[47] Verhulst FC, Koot HM, Van der Ende J (1994). Differential predictive value of parents' and teachers' reports of children's problem behaviors: a longitudinal study. J Abnorm Child Psychol.

[48] Overgaard KR, Aase H, Torgersen S (2014). Continuity in features of anxiety and attention deficit/hyperactivity disorder in young preschool children. Eur Child Adolesc Psychiatry.

[49] Oerbeck B, Overgaard KR, Aspenes ST (2017). ADHD, comorbid disorders and psychosocial functioning: How representative is a child cohort study? Findings from a national patient registry. BMC Psychiatry.

[50] Furu K, Karlstad O, Zoega H (2017). Utilization of stimulants and atomoxetine for attention-deficit/hyperactivity disorder among 5.4 million children using population-based longitudinal data. Basic Clin Pharmacol Toxicol.

[51] Jensen CM, Steinhausen HC (2015). Comorbid mental disorders in children and adolescents with attention-deficit/hyperactivity disorder in a large nationwide study. Atten Defic Hyperact Disord.

[52] Reale L, Bartoli B, Cartabia M (2017). Comorbidity prevalence and treatment outcome in children and adolescents with ADHD. Eur Child Adolesc Psychiatry.

